# Perturbations of the endocannabinoid system in mantle cell lymphoma: correlations to clinical and pathological features

**DOI:** 10.18632/oncoscience.77

**Published:** 2014-09-04

**Authors:** Agata M. Wasik, Lina Nygren, Stefan Almestrand, Fang Zong, Jenny Flygare, Stefanie Baumgartner Wennerholm, Leonie Saft, Patrik Andersson, Eva Kimby, Björn E. Wahlin, Birger Christensson, Birgitta Sander

**Affiliations:** ^1^ Division of Pathology, Department of Laboratory Medicine, Karolinska Institutet, SE 141 86 Stockholm, Sweden; ^2^ Division of Clinical Chemistry, Department of Laboratory Medicine, Karolinska Institutet, SE 141 86 Stockholm, Sweden; ^3^ Division of Hematology, Department of Medicine, Karolinska Institutet, Karolinska University Hospital, SE 141 86 Stockholm, Sweden; ^4^ Clinical Pathology, Karolinska University Hospital Solna, SE 171 76 Stockholm, Sweden; ^5^ Department of Hematology, Stockholm South Hospital, SE 118 83 Stockholm, Sweden

**Keywords:** mantle cell lymphoma, endocannabinoid system

## Abstract

The cannabinoid receptors are upregulated in many types of cancers, including mantle cell lymphoma (MCL) and have been suggested to constitute novel therapeutic targets. The expression pattern of the key members of the endocannabinoid system was analyzed in a well-characterized MCL patient cohort and correlated to biological features. 107 tumor tissues were analyzed for the mRNA levels of cannabinoid receptors 1 and 2 (CNR1 and CNR2) and the two main enzymes regulating the endocannabinoid anandamide levels in tissue: NAPEPLD and FAAH (participating in synthesis and degradation, respectively). NAPEPLD, CNR1 and CNR2 were overexpressed while FAAH expression was reduced in MCL compared to non-malignant B-cells. Both low CNR1 and high FAAH levels correlated with lymphocytosis (p=0.016 and p=0.022, respectively) and with leukocytosis (p=0.0018 and p=0.047). Weak to moderate CNR1 levels were a feature of SOX11 negative MCL (p=0.006). Both high CNR2 and high FAAH levels correlated to anemia (p=0.0006 and p=0.038, respectively). In conclusion, the relative expression of the anandamide synthesizing and metabolizing enzymes in MCL is heavily perturbed. This finding, together with high expression of cannabinoid receptors, could favor enhanced anandamide signaling and suggest that targeting the endocannabinoid system might be considered as part of lymphoma therapy.

## INTRODUCTION

Overexpression of cyclin D1 is a key feature of mantle cell lymphoma (MCL). In the great majority of cases this is due to translocation of the *CCND1* gene on chromosome 11 to the immunoglobulin locus on chromosome 14 t(11;14)(q13;q32) [1]. Recent studies have demonstrated that the lymphoma cells are highly dependent on signals from the microenvironment for their survival and proliferation [2]. Among crucial signaling pathways contributing to MCL pathogenesis are aberrant BCR-signaling and alterations in PI3-kinase, WNT and TGF-beta signaling (reviewed in [3]). Promising new therapeutic agents such as inhibitors of PI3-kinase and Bruton's tyrosine kinase (BTK) interfere with such prosurvival signals [3, 4]. Chemokine receptors are crucial for retaining MCL cells in close contact with stromal cells in the lymphoma niche and may constitute novel targets for therapy [5].

We and others have reported high expression of the G-protein coupled receptors cannabinoid receptor 1 and cannabinoid receptor 2 (encoded by CNR1 and CNR2, respectively) in MCL compared to non-malignant lymphoid tissue or purified non-malignant B-lymphocytes [6, 7]. These receptors bind endogenous lipids, so called endocannabinoids. The cannabinoid receptors, the endocannabinoids and the enzymes regulating the levels of the endocannabinoids comprise the endocannabinoid system (ECS) [8]. Normally, CNR1 is hardly detected in lymphocytes but highly expressed in CNS and regulates synaptic signaling [9]. CNR2 is expressed in the immune system and the receptor protein regulates homing of B-lymphocytes and the architecture of B-cell areas in the spleen [10, 11]. Furthermore, cannabinoid receptor 2, and to lesser extent cannabinoid receptor 1, participate in immune regulation by providing inhibitory or stimulatory signals depending on receptor expression levels, ligand concentrations and cell type [12, 13]. One of the major endocannabinoids is N-arachidonoylethanolamine, also called anandamide. The main enzyme responsible for the biosynthesis of anandamide is N-acyl phosphatidylethanolamine phospholipase D (NAPEPLD), while fatty acid amide hydrolase (FAAH) is the main enzyme metabolizing anandamide. Thus these two enzymes are key components in regulating the cellular anandamide levels.

Important functions of the cannabinoid receptors and endocannabinoid signaling have been described in several types of cancer (astrocytoma, glioma, breast-, prostate-, colon-, pancreatic – and hepatocellular cancer and also non-Hodgkin lymphoma) [13-15]. In general, cancer tissues express higher levels of cannabinoid receptors than the non-malignant counterparts and the endocannabinoid system is therefore considered as a potential novel therapeutic target in cancer therapy (reviewed in [14, 15]).

We have previously shown that exposure of MCL cells to cannabinoids induces cell death *in vitro* [16, 17] and reduces tumor growth in xenograft mouse models [18]. However, hitherto the clinical and biological impact of CNR1 and CNR2 expression in MCL has not been described.

In this study we investigated the expression of CNR1 and CNR2 and the major enzymes involved in the synthesis (NAPEPLD) and metabolism (FAAH) of the endocannabinoid anandamide in a well characterized cohort of MCL patients. The results are correlated to clinical and pathological features.

## RESULTS

### Clinical and pathological features of the MCL cases included

In this study we analyzed the various components of the ECS in MCL diagnostic samples (n=100) and relapse samples (n=7) belonging to the well characterized population-based Stockholm cohort [19]. Lymph node biopsies constituted 81/107 samples. Clinical and pathological features of this cohort are presented in Table 1.

**Table 1:** Clinical and pathological features of the MCL patients included in the study

Clinical and pathological features	All, n=107
Median age (range)	69.2 (32.1-91.7)
Age > 65 (%)	63/107 (58.9)
Sex, male/female	75/32
B symptoms (%)	30/101 (29.7)
ECOG >= 2 (%)	5/102 (4.9)
Nodal presentation > 4 nodal sites (%)	64/102(62.7)
Splenomegaly (%)	44/96 (45.8)
Ann Arbor IV (%)	82/103 (79.6)
WBC > 10×10^9^/L (%)	26/102 (25.5)
Lymphocytes > 5×10^9^/L (%) (leukemic disease)	20/101 (19.8)
High serum LDH (%)	42/100 (42)
MIPI high risk (%)	28/79 (35.4)
Ki67 high >= 30% (%)	41/87 (52.6)
Anemia (Hb < 120g/L)	30/103 (29.1)
SOX11 positivity (%)	78/85 (91.8)
p53 positivity > 20% of cells by IHC (%)	11/83 (13.3)
ASCT first line treatment (%)	26/96 (27.1)
Blastoid morphology (%)	14/90 (15.6)
Indolent disease (%)	14/92 (15.2)

### Characterization of the endocannabinoid system involved in anandamide signaling in primary MCL samples

**Figure 1:**
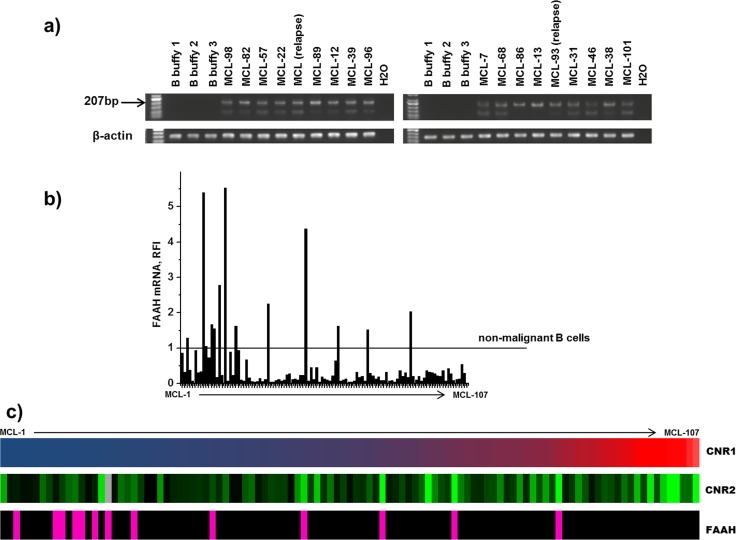
mRNA expression a) NAPEPLD in non-malignant B-cells and MCL analyzed by RT-PCR; b) FAAH mRNA in MCL analyzed by qPCR and expressed as relative fold increase (RFI) compared to non-malignant B cells; c) expression of CNR1, CNR2 and FAAH in MCL Cases are sorted according to increasing levels of CNR1 (navy blue represents the lowest expression, RFI=0.63, red corresponds to the highest expression, RFI=4652.37). For CNR2 black shows the lowest expression RFI=1.06, light green – highest RFI=176.97, grey – data unknown). Cases with FAAH expression higher than in non-malignant B cells from buffy coat 1 (RFI >1) are marked in pink.

Previously, we and others showed that the CNR1 and CNR2 are highly expressed in MCL compared to reactive lymphoid tissues [6, 17] or isolated non-malignant B-cell subpopulations [7]. In the current study, expression of CNR1, CNR2, NAPEPLD and FAAH was measured by PCR and results were normalized to the expression levels in non-malignant B-lymphocytes isolated from peripheral blood to map the expression pattern. Furthermore, we investigated whether the normalized expression values correlated to clinico-pathological data. Expression of NAPEPLD was analyzed by RT-PCR since the expression levels in non-malignant B cells were undetectable and could not be used for normalization. RT-PCR results showed that NAPEPLD was highly expressed in 18/18 (100%) analyzed MCL cases (Figure 1a) but absent in normal B-cells. All other components of the ECS were analyzed by qPCR. In order to analyze the variation in mRNA expression levels and to rank the expression among cases we normalized to one of the peripheral blood samples, purified B-cells from buffy coat 1. The values are expressed as relative fold increase (RFI). Thirteen MCL cases expressed higher FAAH than the control B-cells. Interestingly, in 95/107 (88%) the expression of FAAH was low (Figure 1b). CNR1 mRNA was overexpressed in 105/107 (98%) MCL samples but with a wide RFI range (0.63 to 4652.37) (Figure 1c). CNR2 mRNA was in all patients samples expressed at higher levels than in normal B-cells (RFI range 1,06 to 176,97) (Figure 1c). Expression levels of CNR1 and CNR2 correlated moderately (Spearman's correlation coefficient 0.39, p=0.00005). Of note, in samples with high FAAH expression (12%) the CNR1 expression was significantly lower (p=0.003). The expression levels of CNR1, CNR2 and FAAH did not correlate to the tumor cell content in the tissue as measured by flow cytometry.

### CNR1, CNR2 and FAAH expression in relation to clinical and pathological features

We normalized CNR1, CNR2 and FAAH expression to non-malignant B-cells from a single donor as described above and analyzed the expression levels in relation to clinical features (age, sex, anemia, thrombocytopenia, leukocytosis and lymphocytosis, LDH-levels, Ann Arbor stage, B-symptoms, splenomegaly, nodal presentation, mantle cell lymphoma international prognostic index (MIPI)), to overall survival (OS) and to pathological features (blastoid morphology, proliferation measured as Ki67-index, SOX11 positivity and p53 expression analyzed by IHC).

OS was analyzed in 100 of the cases with ECS expression data in the diagnostic biopsy. For assessing a possible association between gene expression and OS the 100 MCL cases were grouped into two or three groups based on the CNR1, CNR2 or FAAH medians or tertiles of RFI values. Kaplan-Meier analysis showed no significant difference in OS with respect to CNR1, CNR2 nor FAAH expression.

**Figure 2:**
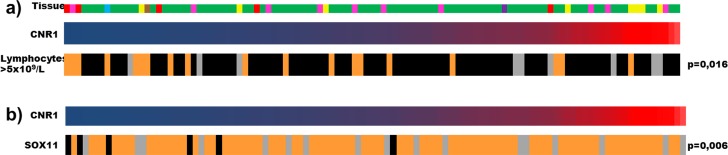
Graphical representation of statistical significance between CNR1 expression levels and other parameters Tissue types in which gene expression was investigated are color-coded: green – lymph node, pink – tonsil, yellow – spleen, brown – bone marrow, purple – gastrointestinal tract, red – blood, blue – pleura. MCL cases were sorted according to increasing levels of CNR1 expression (navy blue shows the lowest expression RFI=0.63, red – the highest RFI=4652.37): a) low levels of CNR1 mRNA correlate to lymphocytosis (lymphocytes >5×10^9^/L), marked in orange, grey color – data unknown; b) CNR1 mRNA levels correlate to immunoreactivity for SOX11. SOX11 positive cases are marked in orange, SOX11 negative in black, grey color – data unknown.

Low CNR1 mRNA expression correlated to lymphocytosis defined as lymphocyte count >5×10^9^/L (p=0.016) (Figure 2a) and to leukocytosis (p=0.0018). In our cohort 84/90 cases were SOX11 positive by IHC. High CNR1 mRNA content was positively correlated to SOX11 positivity (p=0.006) (Figure 2b). We did not find any significant difference between CNR1 expression and other investigated features listed in Table 1.

**Figure 3:**
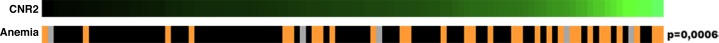
Graphical representation of the statistical significance between high CNR2 levels (blue – lowest expression, RFI=1.06, red – highest RFI=176.97) and anemia (orange color - anemia, grey color – data unknown)

We found a significant association between high CNR2 expression and anemia (p=0.0006) (Figure 3) but not with any of the other clinical and pathological features. High FAAH expression was significantly correlated to lymphocytosis (p=0.022) and leukocytosis (p=0.047), to anemia (p=0.038) and to immunohistochemical p53 positivity (p=0.001).

## DISCUSSION

The ECS has attracted attention as a potential target for therapy in inflammatory disorders and cancer. A number of agents targeting various components of the ECS have been designed and tested in clinical trials [13, 15]. MCL is an aggressive disease with usually poor survival [20] and new therapeutic options are clearly needed. We here analyzed the key components of the ECS and correlated the findings to clinico-pathological characteristics of the patients. Our study shows that the ECS is heavily perturbed in MCL. NAPEPLD was highly expressed in MCL compared to non-malignant B-cells. 88% MCL cases investigated had lower FAAH mRNA levels compared to non-malignant B-cells, suggesting that the malignant cells might accumulate anandamide. In addition, MCL expressed high levels of both cannabinoid receptors, favoring endocannabinoid signaling.

A subset of cases was characterized by high FAAH and weak to moderate CNR1 expression, suggesting that these cases do not have an upregulated endocannabinoid system and may have a different pathophysiology. In line with this both high FAAH and low CNR1 in tumor tissue were seen in patients with lymphocytosis at diagnosis.

Recently, the transcription factor SOX11 was described to be aberrantly expressed in approximately 95% of MCL cases, while normal B cells are SOX11 negative [19, 21-24]. Our study contained mostly SOX11 positive cases, and we found a significant correlation between high SOX11 expression and high expression of CNR1. Among the SOX11 negative MCL 5/6 cases were in the lowest range (lowest 25%) of CNR1 expression. Interestingly, Fernandez et al. described a subset of nonnodal leukemic MCL cases with indolent clinical course, characterized by low SOX11 expression [21]. In their gene expression analysis of SOX11 negative and SOX11 positive MCL, CNR1 was identified as significantly lower in SOX11 negative MCL. In our cohort, we had only 1 case that would correspond to the leukemic, indolent MCL described by Fernandez et al. [21] and in fact, in our study leukemic disease was associated with nodal presentation and correlated to poorer overall survival (p=0.037). In spite of this difference, low CNR1 expression seems to be a feature of lymphocytosis in MCL. MCL survival and proliferation depend on the signals from the tumor microenvironment [2]. Importantly, MCL cells disrupted from the lymphoid/bone marrow microenvironment lose pro-survival support from stromal cells [2, 25]. Our data indicate that lymphocytosis in MCL is characterized by low CNR1 expression, implying that high CNR1 is associated with homing to lymphoid tissue, increased adhesion and/or reduced egress of MCL cells from lymph nodes. Signaling through CNR1 might thus be involved in regulating the retention of MCL cells in lymphoid tissue a niche that provides optimal conditions for tumor cell growth and survival and potentially enhanced resistance to many therapeutic agents. The possible involvement of CNR1 in leukocyte homing is supported by several experimental studies in which CNR1 stimulation altered adhesive properties. Kianian et al. showed recently that CNR1 inhibition by specific antagonist AM281 reduced leukocyte recruitment within the intestinal microvasculature in experimental endotoxemia in Lewis rats [26]. CNR1 has further been suggested to be involved in adhesion of T cells [27] and synovial fibroblasts [28] in experimental disease models.

We found that all MCL cases expressed higher levels of CNR2 than non-malignant B-cells. A significant correlation between high CNR2 expression and anemia was found. As the cannabinoid receptor 2 is responsible for retention of immature B cells in the bone marrow [29] and leukemic cells in the bone marrow disrupt the normal hematopoiesis [30], we hypothesized that the anemia might be secondary to high bone marrow infiltration but morphological re-evaluation of staging bone marrow biopsies did not support this hypothesis (data not shown).

In summary, this study investigated the expression of cannabinoid receptors and enzymes regulating levels of the endocannabinoid anandamide in diagnostic samples of MCL. MCL has high expression of a key enzyme in the anandamide synthesis, NAPEPLD and mostly low expression of the anandamide metabolizing enzyme FAAH, suggesting that high anandamide levels are favored in this lymphoma subtype. In addition, the expression of both cannabinoid receptors was upregulated in most cases as compared to non-malignant B-cells. In contrast, only a few cases were characterized by low CNR1 expression and high expression of FAAH and these MCL had elevated lymphocyte count at diagnosis. Our data point to a role for the ECS in homing, adhesion and egress of MCL cells from the lymphoid tissue. Importantly, the ECS is druggable and ligands to CB1 and CB2 are used in clinical trials for various diseases including multiple sclerosis and cancer [15, 31-33]. This suggests that targeting the endocannabinoid system might be a possible novel treatment modality in MCL.

## MATERIALS AND METHODS

### Patients and clinical data

Diagnostic samples with available frozen tissue from patients diagnosed with MCL between January 1994 and March 2013 at the Pathology Department of Karolinska University Hospital were retrieved. In total, 107 MCL cases were identified and 100 of these were samples taken at diagnosis, before start of treatment. These patients are part of a heterogeneously treated, unselected, populationbased cohort [19]. Baseline and follow-up clinical data were obtained from hospital files.

### Tumor tissue, and immunohistochemical analysis

Tumor tissue from lymph nodes (n=81), spleens (n=8), tonsils (n=10) or gastrointestinal biopsies (n=1) removed for diagnostic purposes were snap frozen and stored at – 80oC until use. In some instances cell suspensions from bone marrow (n=1), blood (n=5) or pleural fluid (n=1) were retrieved by Ficoll separation (Ficoll-Paque PLUS, GE Healthcare), viability frozen in DMSO and stored at – 150oC.

Lymphomas were diagnosed according to the WHO classification [1]. All cases were cyclin D1 positive by immunohistochemistry (IHC) and/or positive for t(11;14) (q13;q32) translocation by interphase FISH or cytogenetic analysis. IHC staining for cyclin D1, Ki-67, p53 and SOX11 was done on whole sections of paraffin embedded diagnostic biopsies as previously described [19].

### RNA isolation and cDNA synthesis

RNA was isolated using the RNeasy Plus mini kit (Qiagen) according to the manufacturer's protocol. Quantification and quality of the RNA preparations were measured using the NanoDrop ND-1000 spectrophotometer (Saveen Werner). Complementary DNA (cDNA) was synthesized using the Omniscript Reverse Transcription (RT) kit (Qiagen) according to manufacturer's protocol. RNaseOut Recombinant Ribonuclease inhibitor and the Oligo dT primers used were purchased from Invitrogen.

### Quantitative PCR (qPCR)

mRNA expression levels of CNR1, CNR2, NAPEPLD and FAAH were assessed by Real-Time PCR using Platinum SYBR Green qPCR Supermix-UDG (Invitrogen) according to the manufacturer's protocol. Primers sequence (Eurogentec) for the selected genes were as follows: CNR1 forward: 5-CATTAAGACGGTGTTTGCATTCT-3, reverse: 5-CGTGTCGCAGGTCCTTACTC-3; CNR2 forward: 5 – GACACGGACCCCTTTTTGCT-3, reverse 5 – CCTCGTGGCCCTACCTATCC-3; NAPEPLD forward: 5-CGGAGCCTGCATCTCTGAAG-3, reverse: 5-AGGATATTGGCTGCTTGTCATCA-3; FAAH forward: 5-GAAGTCTCGTTCGGCTGGAA-3, reverse: 5-ACTGGGCAATCACGGTTTTG-3; β-actin forward: 5-AAAGACCTGTACGCCAACACA-3, reverse: 5-AGTACTTGCGCTCAGGAGGA-3. Each sample was prepared in triplicates in a 96 well plate (BioRad) and the reactions were performed with the C1000 Thermal cycler (BioRad). An initial step was performed at 95°C for 2 min, followed by 40 cycles of 95°C for 15 seconds and finished by 57°C for 30 sec. The results were analyzed and cycle threshold (Ct) values of transcripts were quantified using CFX manager software (BioRad). The ΔCt values were calculated using β-actin as reference. Expression levels of CNR1, CNR2 and FAAH of mRNA were normalized to the expression levels of the respective gene in the B-cells isolated from the buffy coat 1 (used as a standard). NAPEPLD mRNA in B-cells from buffy coats from three different healthy donors was undetectable and thus the PCR amplification products for NAPEPLD were run on the 1.5% agarose (Gibco) gel. The image was taken using FluorChem SP (Alpha Innotech). Two bands of the NAPEPLD PCR product were cut out from the gel, DNA was extracted using kit from Fermentas, DNA was precipitated using Pellet Paint NF Co-precipitant from Novagene and sent off for sequencing to KIGene to confirm that the band at 207bp represented NAPEPLD.

### Statistical analysis

Overall survival (OS) was calculated from the date of MCL diagnosis to the date of death. Associations with survival were evaluated using Kaplan–Meier curves and the log-rank test. Statistical analysis included Spearman rank correlation and Mann-Whitney test. A p value of <0.05 was considered significant.

### Ethical permission

The Regional Central Ethical Review Board at Karolinska Institutet has approved the research and given all necessary ethical permissions.
